# Supplementary material for: Structures of the DarR transcription regulator reveal unique modes of second messenger and DNA binding
Source: Nat Commun. 2023 Nov 9;14:7239. doi: 10.1038/s41467-023-42823-0 (PMC10636190; doi:10.1038/s41467-023-42823-0)
Supplement: Supplementary file 1 — Supplementary information [file 41467_2023_42823_MOESM1_ESM.pdf]

SUPPLEMENTARY INFORMATION

Structures of the DarR transcription regulator reveal unique modes of second messenger and DNA binding

Maria A. Schumacher*, Nicholas Lent, Vincent Chen, Raul Salinas

Department of Biochemistry, Duke University School of Medicine, Durham NC 27710, USA

Corresponding author: Maria.schumacher@duke.edu

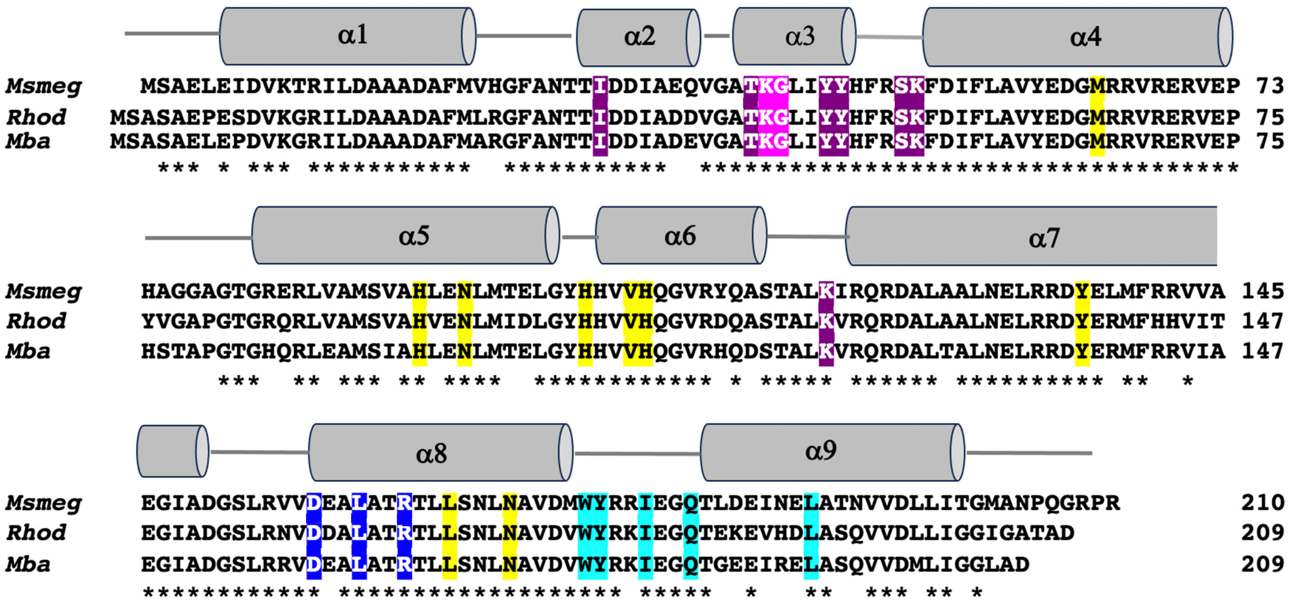

Supplementary Figure 1. Multiple sequence alignment of the three DarR homologs used in this study. Shown above the alignments are the secondary structural elements obtained from the structures. Completely conserved residues are indicated by an asterisk below the alignment. Residues that participate in non-specific DNA contacts are highlighted in violet. Residues making base specific contacts are pink and residues that interact with the ligand in the effector binding pocket are colored yellow. Residues that contact adenine nucleotides are colored cyan (contacts from one subunit) and dark blue (contacts from the other subunit to the c-di-AMP).

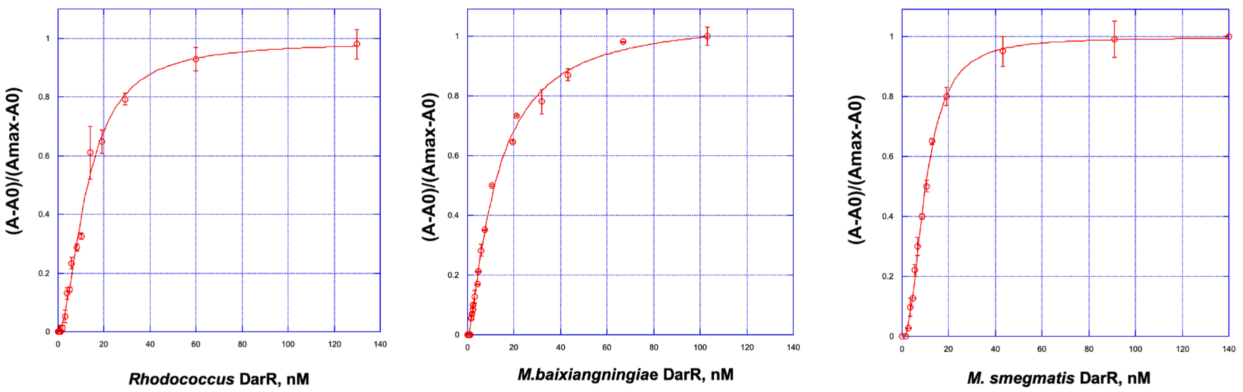

Supplementary Figure 2. FP binding isotherms of DarR homolog interaction with DNA. The FP isotherms are of *Rhodococcus* sp. USK13 DarR, *M. baixiangningiae* DarR and *M. smegmatis* DarR binding to the fluoresceinated 20 bp operator site in HEPES buffer (25 mM HEPES pH 7.5, 150 mM NaCl, 5% (v/v) glycerol). The x and y axes are DarR concentration in nM and normalized mP ((A-A_0)/(A_{max}-A_0)), respectively. The K_ds are 11.4 ± 1.2 nM, 12.4 ± 0.7 nM and 10.1 ± 1.0 nM, respectively. Data points represent mean values ± SD with the error bars centered at the mean. The error in overall K_d was determined as the SD between the calculated K_ds for three runs for each protein tested.

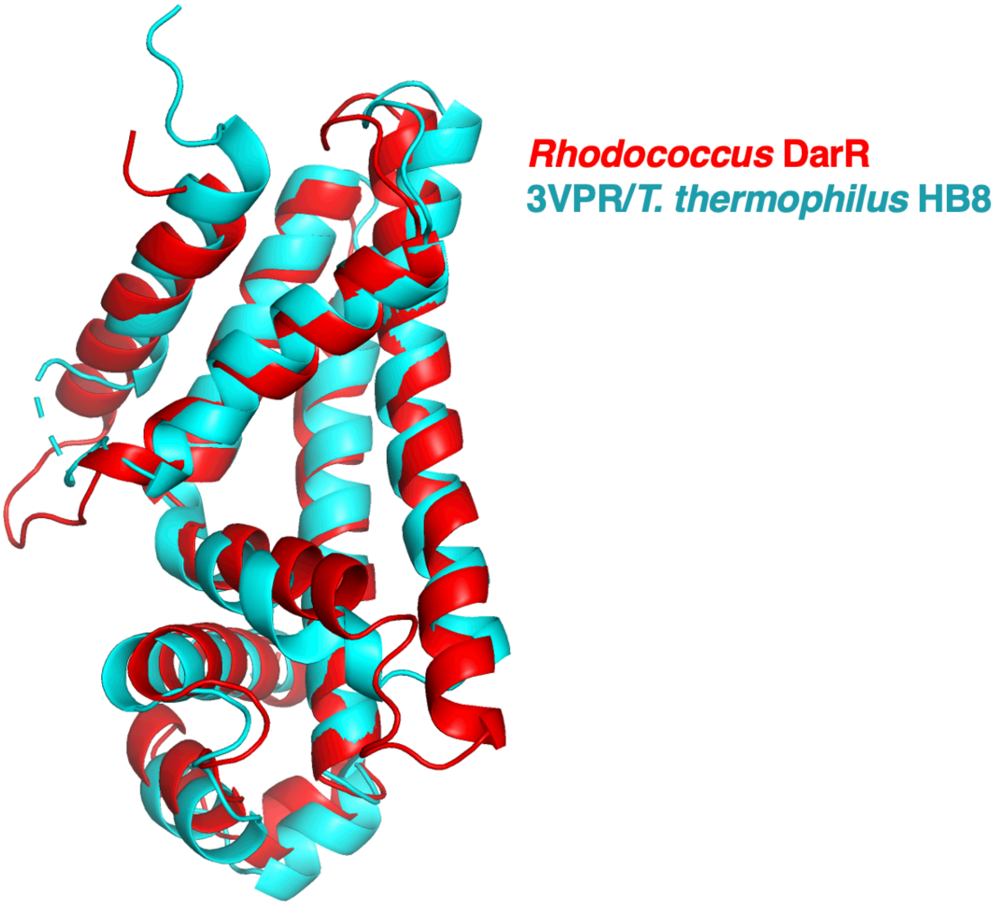

Supplementary Figure 3. Superimposition of the *Rhodococcus* sp. USK13 DarR (red) and *T. thermophilus* HB8 PfmR (3VPR [http://doi.org/10.2210/pdb3VPR/pdb]) (cyan) subunit structures. 160 Cα atoms of each structure can be overlaid with an rmsd of 1.9 Å.

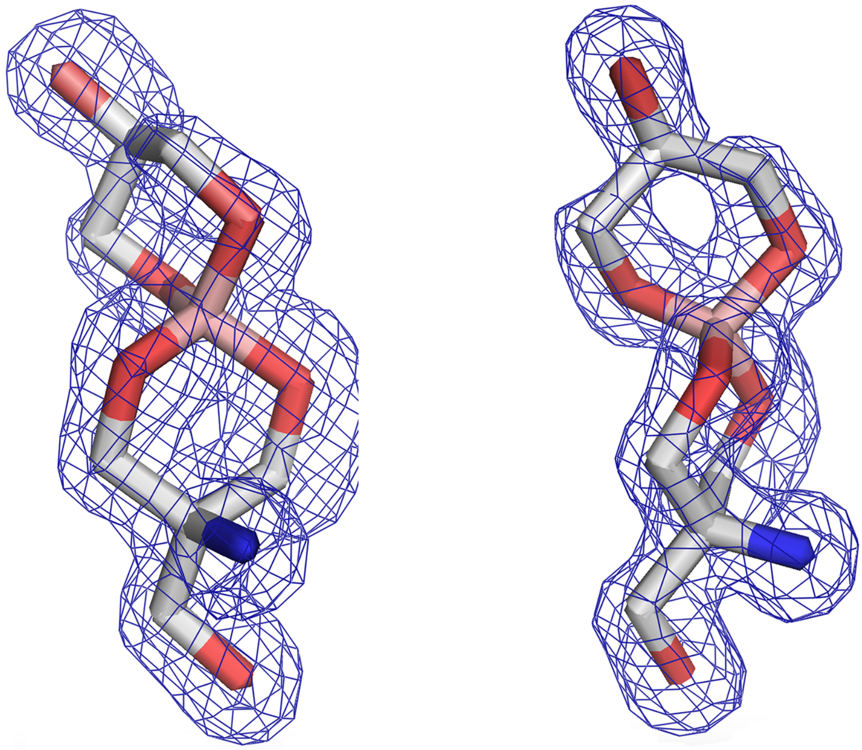

Supplementary Figure 4. **Electron density for the fortuitously bound ligand in the *M. baixiangningiae* DarR structure**. The figure shows two views of the omit mF_o-DF_c electron density (blue mesh) for the ligand contoured at 3.5 σ. The Sigma-A weighted omit map was generated by removing the ligand and performing 30 cycles in Phenix_refine.

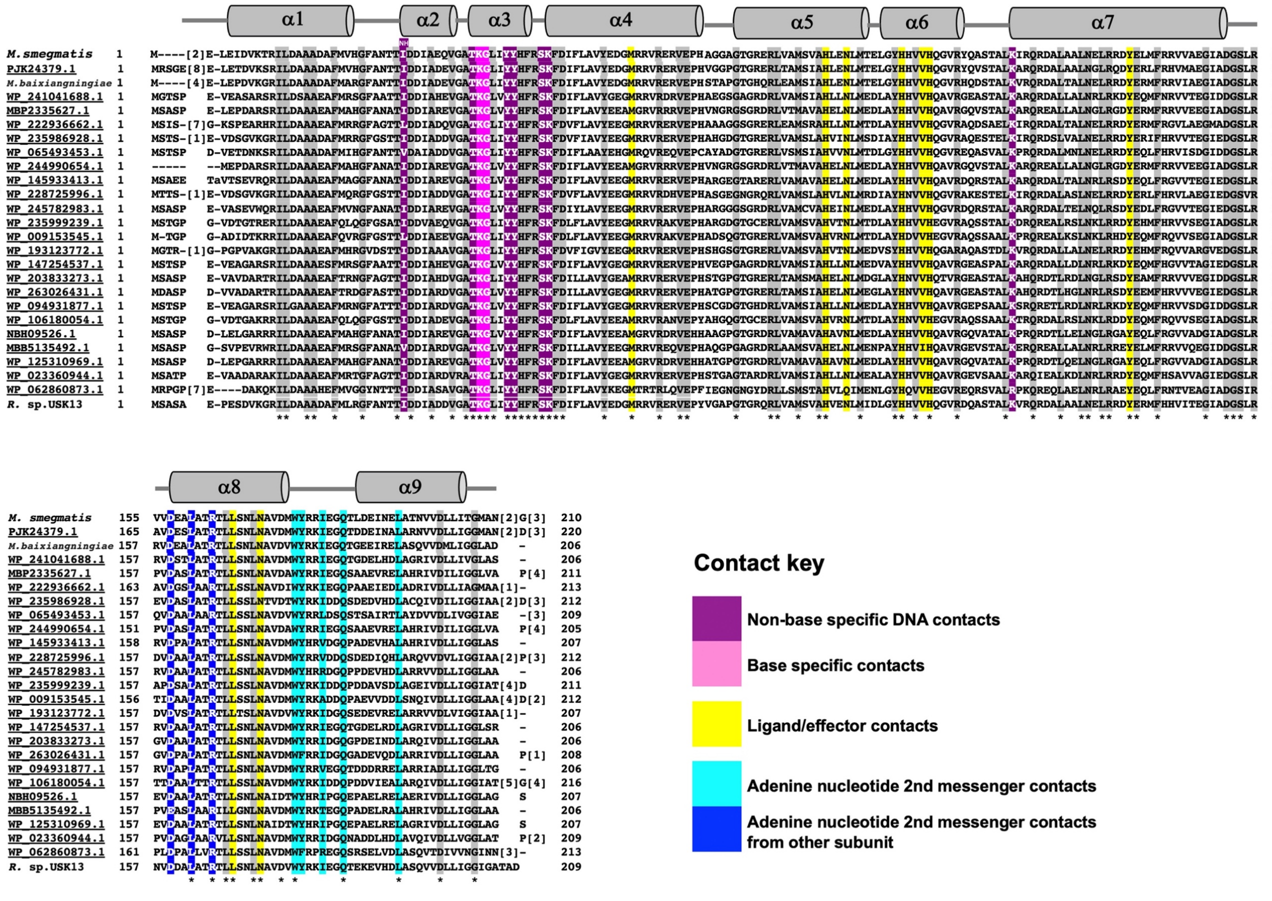

Supplementary 5. Multiple sequence alignment of DarR homologs. Shown above the alignments are the secondary structural elements from the structures. Conserved residues are highlighted in grey and indicated by an asterisk below the alignment. Residues that participate in nonspecific DNA contacts are highlighted in violet. Residues making base specific contacts are pink, residues that interact with the ligand in the effector binding pocket are colored yellow. Residues that contact the adenine nucleotides are colored cyan (contacts from one subunit) and dark blue (contacts from the other subunit). The TetR family (DarR homologs) proteins in the alignment are from the following organisms: *M. smegmatis*: *Mycolicibacterium smegmatis* MC2 155 (included in this study), PJK24379.1: *M. goodii*, *M. baixiangningiae: Mycolicibacterium baixiangningiae* (included in this study), WP_241041688.1: *Pseudonocardia alaniniphila*, MBP2335627.1: *Saccharothrix coeruleofusca*, WP_222936662.1: *Streptomonospora* sp. PA3*,* WP_235986928.1: *Halosaccharopolyspora lacisalsi,* WP_065493453.1: *Rhodococcus opacus,* WP_244990654.1: *Saccharothrix coeruleofusca*, WP_145933413.1: *Amycolatopsis bartoniae*, WP_228725996.1: *Prauserella sediminis,* WP_245782983.1: *Amycolatopsis sacchari*, WP_235999239.1: *Qaidamihabitans albus,*WP_009153545.1: *Saccharomonospora marina*, WP_193123772.1: *Nocardiopsis coralli*, WP_147245437.1:*Pseudonocardia hierapolitana*, WP_203833273.1: *Actinoplanes regularis,* WP_263026431.1: *Actinoplanes* sp. KI2, WP_094931877.1: *Pseudonocardia* sp. MH-G8, WP_106180054.1: *Prauserella shujinwangii*, NBH09526.1: *Amycolatopsis* sp. SID8362*,* MBB5135492.1: *Thermocatellispora tengchongensis*, WP_125310969.1: *Amycolatopsis eburnea*, WP_023360944.1: *Actinoplanes friuliensis*, WP_062860873.1: *Brevibacterium linens*, *R.* sp. USK13: *Rhodococcus* sp. USK13 (included in this study).

```
Msmeg  MAVKSGARSGRNVVHLARPTLRETLGRLAPGTPLRDGLERILRGRTGALIVLGYDDSVEAICDGGFVLDV
Rhod              MNDAESSSALRETIARLAPGTALRDGLERILRGRTGALIVLGYDEQMEEICDGGFELDV
Mba    -MAVKTARTSSNVVQLARPTLRETLGRLAPGTPLRDGLERILRGRTGALVVLGYDDSVEAICDGGFELDV
                           ****  ****** **************** *****   * **********

Msmeg  RYAPTRLRELSKMDGAVVLSSDGSRILRANVQLVPDPSIPTDESGTRHRSAERTAIQTGYPVISVSHSMS
Rhod   EFAPTRLRELSKMDGAVVLSTDGSRIVRANVQLVPDHKIPTVESGTRHRAAERTAMQTGYPVVSVSQSMS
Mba    RYAPTRLRELSKMDGAVVLSSDGTRILRANVQLVPDPSIPTDESGTRHRSAERTAVQTGYPVISVSHSMS
         ****************** ** ** *********  *** ******* ***** ****** *** ***

Msmeg  IVTVYVAGERHVVPDSATILSRANQTIATLERYKGRLDEVSRQLSTAEIEDFVTLRDVMTVVQRLEMVRR
Rhod   IVSVYVGGIRHVIDGSATILSRANQAVATLERYKARLDEVTRQLSVVEIEDFVTLRDALTVVQRLEMVRR
Mba    IVTVYVAGERHVVPDTPTILSRANQTIATLERYKSRLDEVSRQLSTAEIEDFVTLRDVMTVVQRLEMVRR
       ** *** * ***     ********  ******* ***** ****  **********  ***********

Msmeg  ISLEIDADVVELGTDGRQLKLQLDELVGDNETARELIVRDYHAYPDPPTAAQVAATLEELDSLSDSELLD
Rhod   VSVEIEQDVLELGTDGRQLALQLEELVGDNDVARELIVRDYLAGSGPAPAPDVEKSLTALDKITDADLLD
Mba    ISLEIDADVVELGTDGRQIKLQLEELVGDNDNARELIVRDYHANPDPPTPAQVSATLEELDSLSDNELLD
        * **  ** ********  *** ******  ********* *   *     *   *  **   *  ***

Msmeg  FTVLARVFGYPSTAEAQDSAMSSRGYRAMAAIPRLQFAHVDLLVRSFGSLQNLLAASADDLQSVDGIGSM
Rhod   LTTLARAFGYPGTIEALEAPMSPRGYRVLTRVPRLQFNQIHRLVGSFGTLQSLLAATAADLQSVEGIGGL
Mba    FTALARVFGYPSTLEAQDSAMSSRGYRAMAGIPRLQFAHVDLLVRSFGSLQGLLAASADDLQSVDGIGSM
        * *** **** * **    ** ***      *****     ** *** ** **** * ***** ***

Msmeg  WARHIREGLSLLAESTIADRLA
Rhod   WARHIREGLSRLAETSISGPYD
Mba    WARHIREGLSLLAESTIADRLA
       ********** ***   *
```

Supplementary Figure 6. **Multiple sequence alignment of DisA homologs**. Shown are the DisA (c-di-AMP synthesizing enzymes) proteins in *M. smegmatis* (WP_011731023.1), *Rhodococcus* sp. USK13 (WP_109326508.1) and *M. baixiangningiae* (WP_197375332.1). The *Rhodococcus* sp. USK13 and M. *baixiangningiae* DisA homologs each share 93% identity with the *M. smegmatis* DisA. Residues that are identical in all homologs are indicated by asterisks under the alignment. Highlighted in yellow are residues shown to be key in substrate binding and/or catalysis for the *M. smegmatis* enzyme[1], which are conserved in the homologs from *Rhodococcus* sp. USK13 and *M. baixiangningiae*.

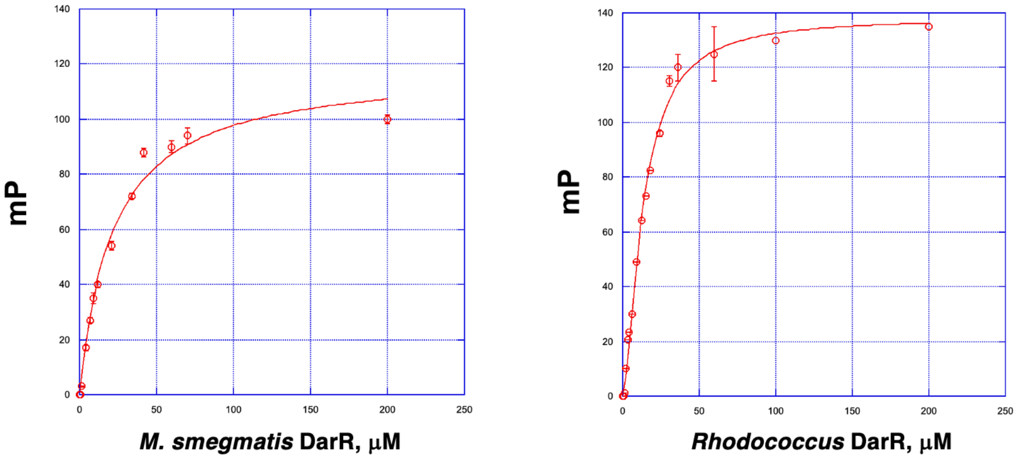

Supplementary Figure 7. FP isotherms showing binding of WT *M. smegmatis* DarR and *Rhodococcus* sp. USK13 DarR to F-c-di-AMP. The binding buffer was 25 mM HEPES pH 7.5, 150 mM NaCl, 5% (v/v) glycerol. The x and y axes are DarR concentration in nM and mP, respectively. The K_ds are 21.6 ± 2.5 μM and 21.9 ± 2.7 μM, respectively. Data points represent mean values ± SD with the error bars centered at the mean. The error in overall K_d was determined as the SD between the calculated K_ds for three runs for each protein.

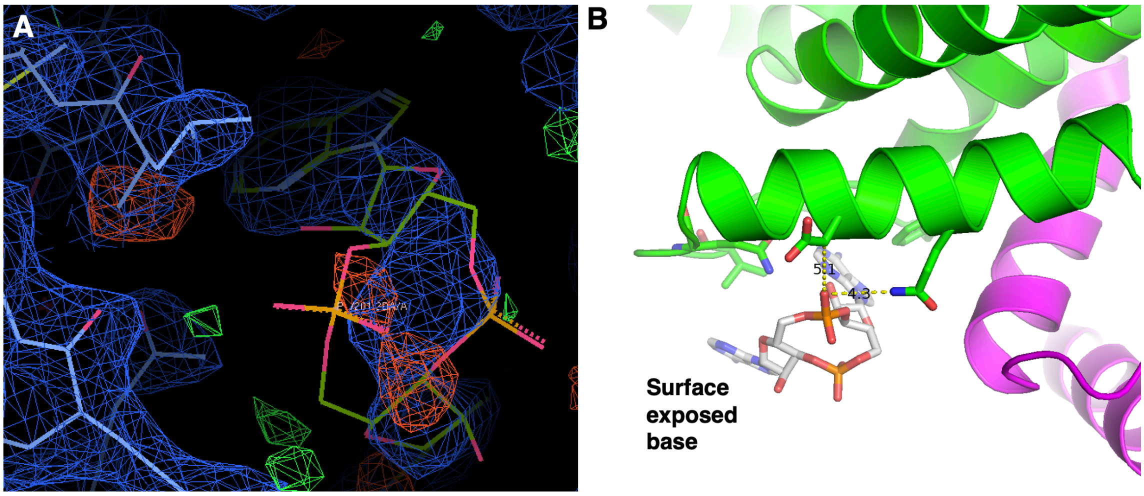

Supplementary Figure 8. **The DarR-bound c-di-AMP phosphates are located on the solvent exposed surface of the structure**. **a** Shown is DarR-c-di-AMP after refinement including the c-di-AMP with electron density included. Blue mesh (contoured at 0.8 σ) is a sigma-A weighted $2mF_o$-DF_c map, red is the positive mF_o-DF_c difference map (contoured at 3 σ) and red is the negative mF_o-DF_c difference map (contoured at 3 σ). **b** Modelling of c-di-AMP bound to DarR. Note the surface exposed nature of one of the bases and phosphate moieties. The closest contacts to the phosphates from DarR are greater than 4 Å.

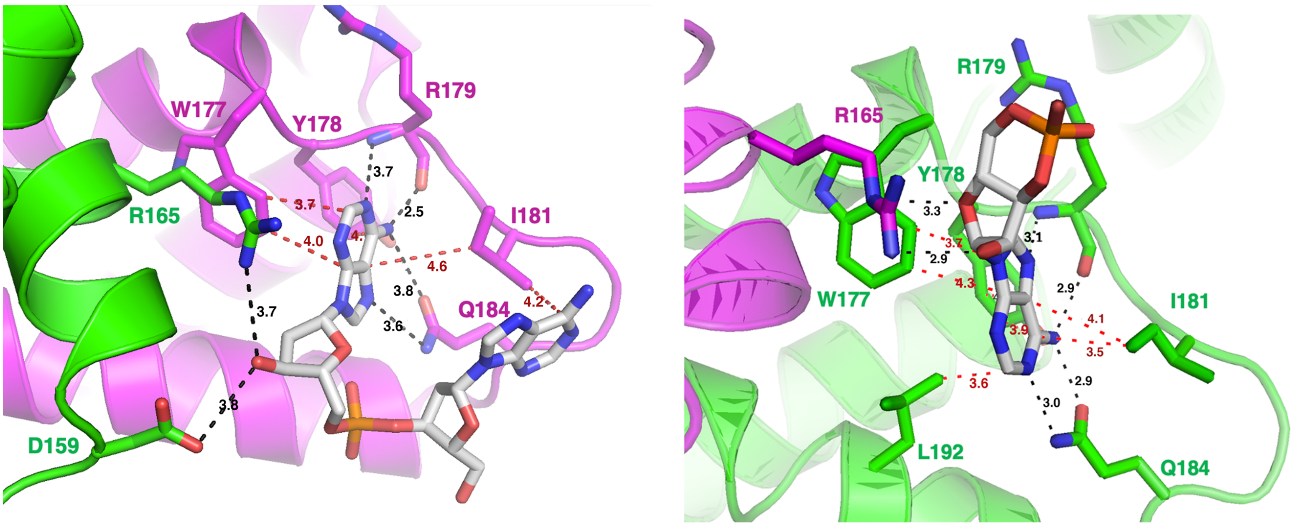

Supplementary Figure 9. **Close-up of DarR-c-di-AMP (left) and DarR-cAMP (right) interactions**. Hydrogen bond interactions are shown as black dashed lines with black labels indicating distances between atoms making the hydrogen bonds. Van der Waals contacts are indicated by red dashed lines with distances included between atoms labeled and colored red.

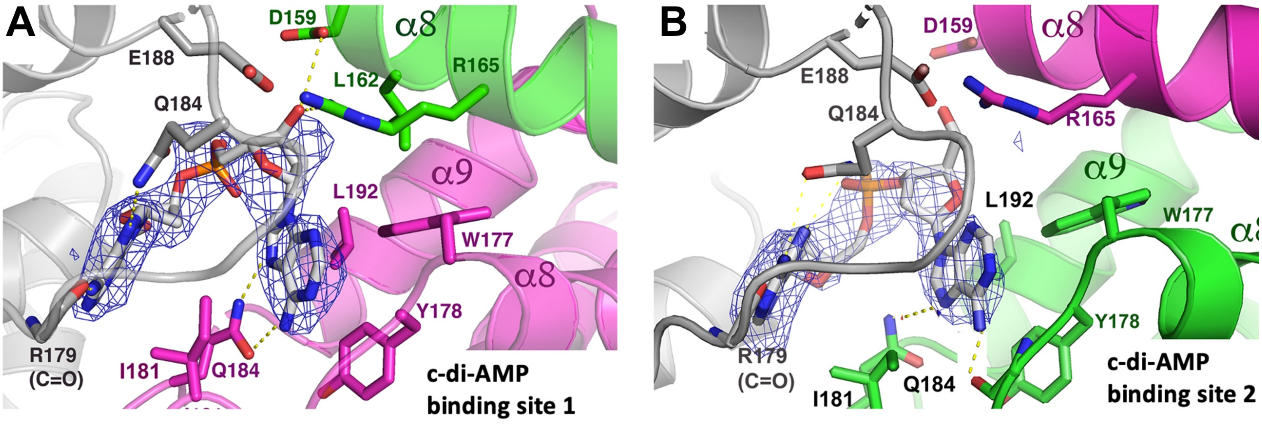

Supplementary Figure 10. Close-up of the two c-di-AMP nucleotide binding interactions. The omit sigma-A weighted mF_o-DF_c electron density, calculated after removing the c-di-AMP molecules prior to refinement, is included as a blue mesh and contoured at 2.9 σ. One DarR subunit is colored green and the other, magenta. Residues that participate in nucleotide binding are shown as sticks and labeled. The subunits of the dimer that interacts with the c-di-AMP molecules are colored green and magenta and the DarR molecules that interact with the exposed base at each site, due to crystal contacts, are colored grey with contacting residues shown as sticks. Shown are the nucleotide binding in each subunit (**a** and **b**).

```
WP_213573200.1,       MSEDP---ADDSAAELDAASESRQDKVLGWVGALNRRPQLIEGLRRVRRALPGDPAFGDP     57
AWT54739.1            ---------------------------MGWLQSANRSPGVVAAVRRFRRALPGDPEFGDP     33
WP_193047576.1        MEVESFGERSGGAEDAEQAPSRGSAGPLGWLYSANHSPGVVEFLRRARRALPGDPDFGDP     60
                                                 :**: : *: * ::  :** ******** ****

WP_213573200.1,       LSTAGPGTARAVARVADRFLDHQPGASREMSLGALQVWQALLERTGRGRGTQEVTIVFTD     117
AWT54739.1            LSVAGDGGPRAAARVADRLLERD-AASREVSLGALQVWQALTERVSGRPANREVTLVFTD     92
WP_193047576.1        LSADGVGGPRAAARVADRLLDRE-AVSREVSLGALQVWQALTERVSGKPANPEVTLVFSD     119
                      **. * *  **.******:*::: ..***:*********** **..   .. ***:**:*

WP_213573200.1,       LVGFSSWSLPAGDTATLALLRDVAKAIETPMVDRGGHVVKRMGDGVMAVFPSPDRAIDAV     177
AWT54739.1            LVGFSSWSLRAGDDATLRLLRRVAQVAEPPLLEAGGHIVKRMGDGMMAVFGDPATAVRAV     152
WP_193047576.1        LVGFSSWSLSAGDDATLRLLRRVAQVFEPPLLEAGGRIVKRMGDGSMVVFTDATTAVRAV     179
                      ********* *** *** *** **:. * *::: **::******* *.** .   *: **

WP_213573200.1,       FAAQDALRNVEVDGYRPRMRVGIHTGVPRQLGSDWLGVDVTIAARMMELGGDGNVMASSA     237
AWT54739.1            LVALDAVKGIEVDGYTPRMRVGIHTGRPQRIGSDWLGVDVNITARVMERAAKGGLMVSHA     212
WP_193047576.1        LTAMNAVRAIEVDGYNPRMRVGVHTGRPQRIGSDWLGVDVNIAARVMERATRGGLVVSQT     239
                      :.* :*:: :***** ******:*** *:::*********.*:**:** .  *.::.* :

WP_213573200.1,       TLSALQPGTLEELGISVKPWRRAFFAPAPSGVPSDLGIWRLRL-----------------     280
AWT54739.1            TLAGIADGELAELGVTVKRERRQLFSARPDGVPDDLVMYRVRTPSRLPAARPREQGPPDA     272
WP_193047576.1        TLDRVAAEDLEALDVTVKRQRRQVFSLKPDGVPPDLSMYRLRRRRPLDADSADDGS----     295
                      **  :    *  *.::**  ** .*:  *.*** ** ::*:*
```

Supplementary Figure 11. **Multiple sequence alignment of adenylyl cyclase homologs from *M. smegmatis*, *Rhodococcus* sp. USK13 and *M. baixiangningiae***. The enzymes are from the following bacteria: WP_213573200.1, *Rhodococcus* sp. USK13; AWT54739.1, *M. smegmatis*; WP_193047576.1, *M. baixiangningiae.* Completely conserved residues among the proteins are indicated by asterisks, highly conserved residues by a double dot and conserved residues by a single dot.

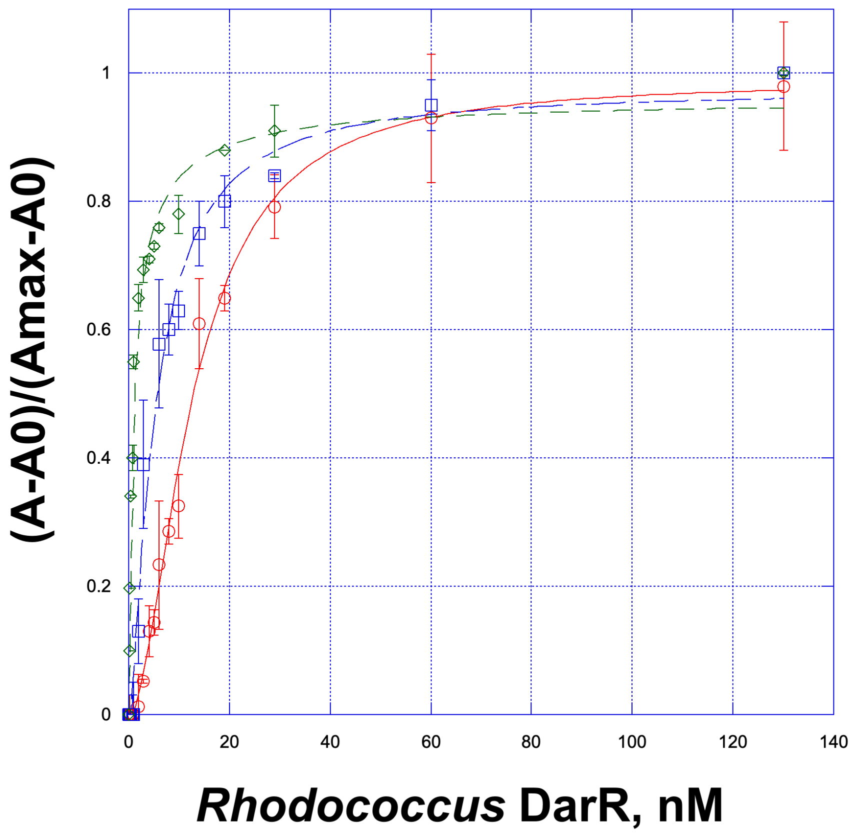

Supplementary Figure 12. Effect on DarR-DNA binding by the presence of adenine nucleotide second messengers. Shown are the binding isotherms comparing binding of *Rhodococcus* sp. USK13 DarR to 20 bp DNA operator site in the absence (red open circles) and presence of 1 mM c-di-AMP (blue open squares) and 1 mM cAMP (green open diamonds). The buffer used was 25 mM HEPES pH 7.5, 150 mM NaCl, 5% (v/v) glycerol. The x and y axes are DarR concentration in nM and normalized mP ((A-A0)/(Amax-A0)), respectively. Data points represent mean values ± SD with the error bars centered at the mean. The error in overall K_d for each was determined as the SD between the calculated K_ds for three runs.

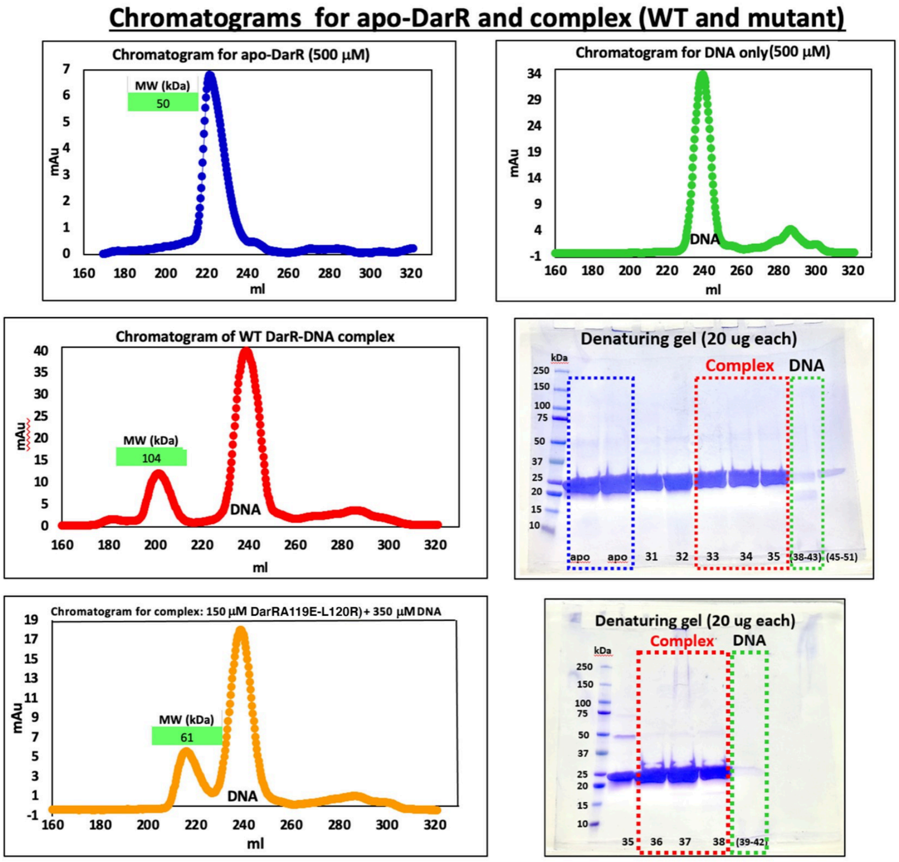

Supplementary Figure 13. Size exclusion chromatography (SEC) analyses of *Rhodococcus* sp. USK13 DarR in the absence and presence of operator DNA. Top panels show SEC elution profile obtained when running the protein alone (500 µM; left), which produced one peak with a predicted MW of ~50 kDa and the DNA only (500 µM) (right). The middle profile is that obtained for the DarR-DNA complex, which produced a MW consistent with a DarR dimer-of-dimers on DNA. Shown on the right are the fractions from each of the peaks that are boxed after concentration and run on an SDS denaturing gel. The last panel shows the elution obtained for the DarR(A119E-L120R)-DNA complex (using a mixture of 150 µM DarR to 350 µM double stranded DNA). Shown to the right of the protein-DNA profiles are the fractions from each of the peaks concentrated and run on a denaturing gel.

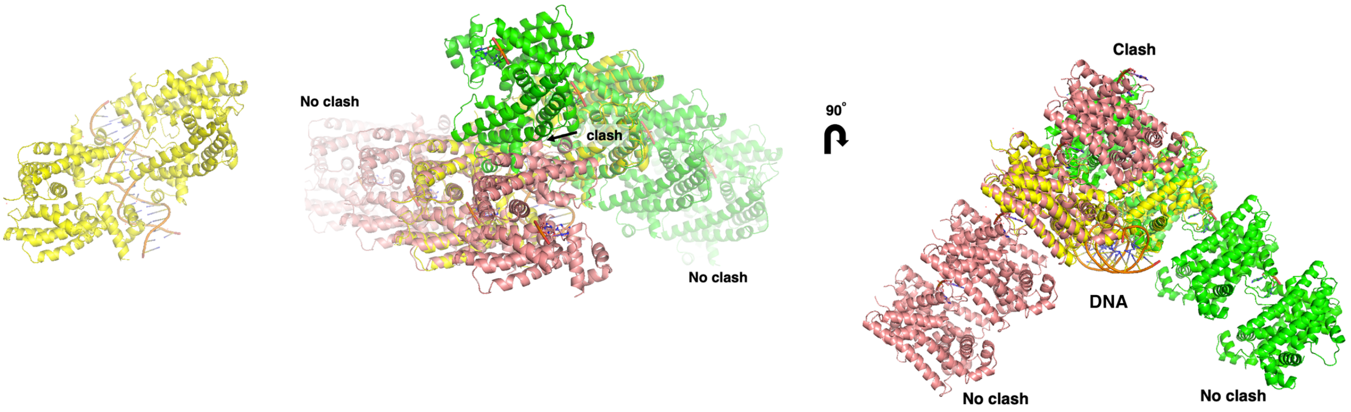

Supplementary Figure 14. Modeling of c-di-AMP mediated DarR polymers on DNA-bound DarR. The c-di-AMP mediated polymers, colored salmon and green, that are observed in the crystal packing of the DarR-c-di-AMP structure are overlaid onto the DNA-bound structure (colored yellow). The figure shows that the central polymers would clash and hence could not form. Possible polymers extending from the outside subunits are angled away from the DNA and hence likely would not significantly impact DarR repression function.

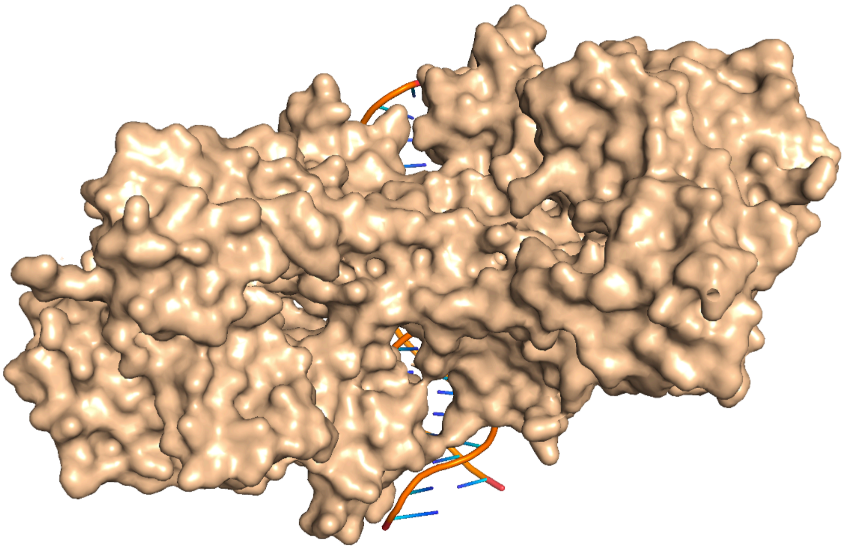

Supplementary Figure 15. DarR dimer-of-dimers encases bound DNA. Structure of the *Rhodococcus* sp. USK13 DarR-DNA complex showing the protein as a tan surface and the DNA as ribbons. Note, binding of the interacting dimer-of-dimers leads to a complete coverage of an entire face of the DNA.

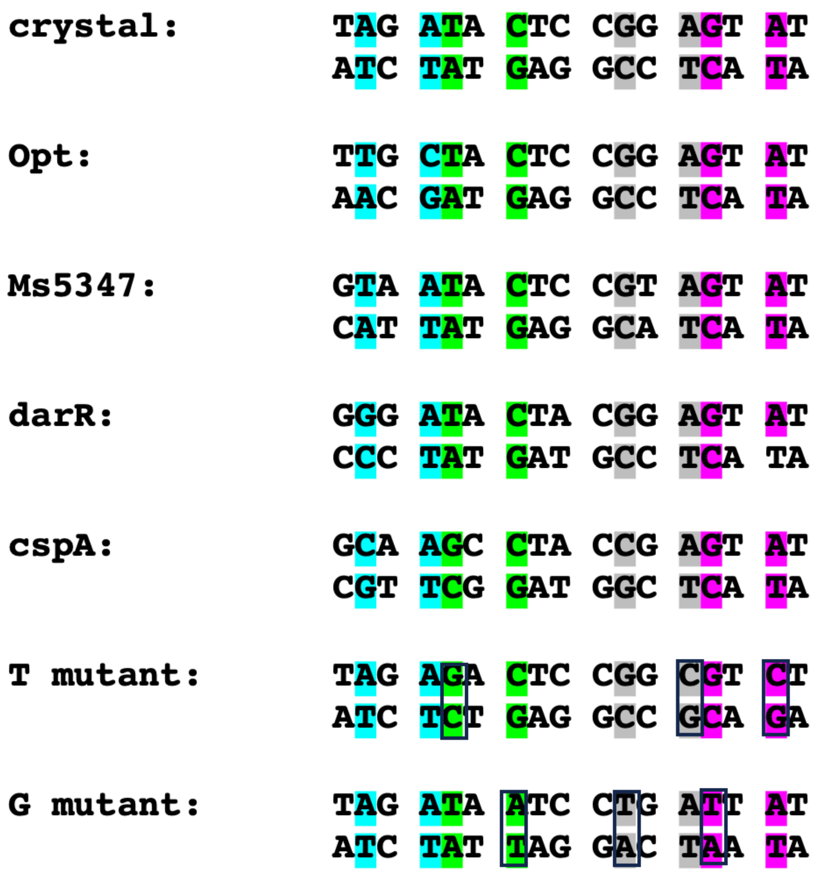

Supplementary Figure 16. **DriD operator sites**. Shown are the consensus regions of the operator sequences used in crystallization experiments, generation of the optimal site, the *in vivo* DarR operator sites from the Ms5347, *darR* and *cspA* promoters and operators from the mutant sites used in FP binding assays. The three TXC motifs in the operator are indicated in green, magenta and grey. Generation of the optimal site was accomplished by adding a fourth TXC motif, which is colored blue. The specific bases mutated in the T and G mutants are boxed. Note, the *darR* and Ms5347 operators all have the third motif.

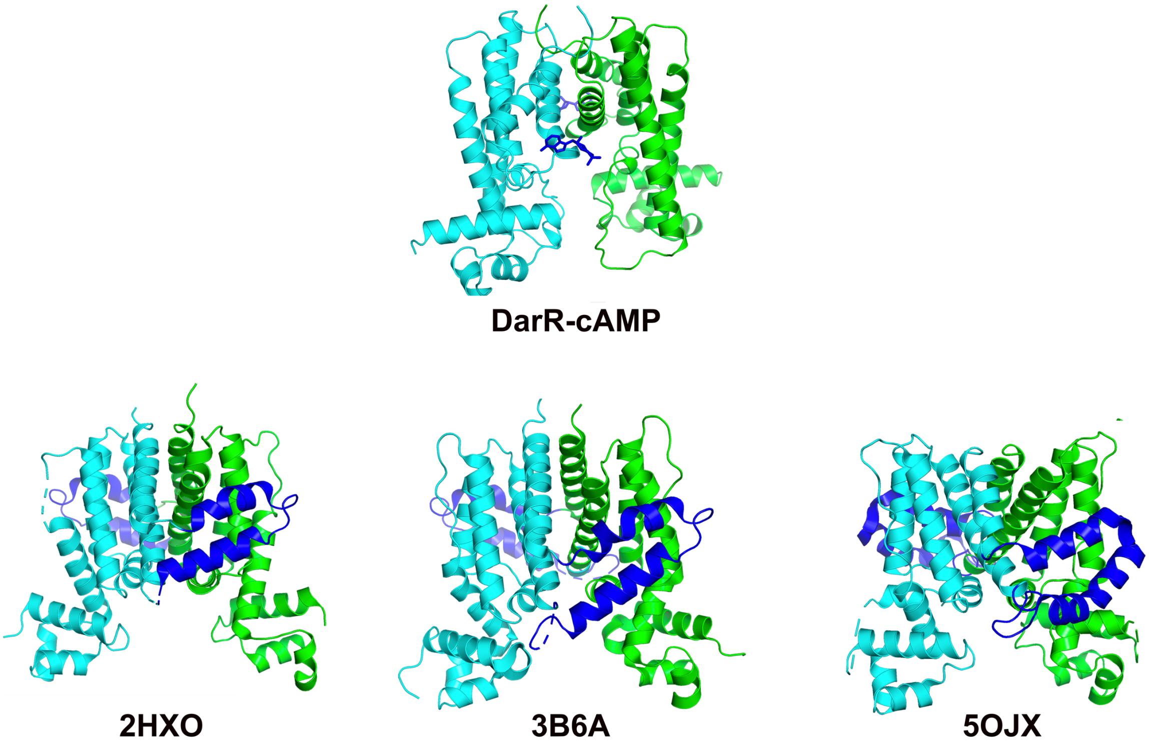

Supplementary Figure 17. Comparison of the dimer of the DarR-cAMP complex to TFR dimers with α8-α9 inserts. Shown top is the DarR dimer cAMP structure with one subunit colored cyan and the other green. The cAMP molecules are shown as sticks and colored blue. Below shows three TFR members that have inserted regions between α8 and α9, which form dimer stabilizing interactions.

Supplementary Table 1: Data collection and refinement statistics: apo DarR and DarR ligand structures

	M.*baixiangningiae* DarR-Tris-boron-glycerol	Apo *M. smeg.* DarR	*Rhodococcus sp.* USK13 DarR-c-di-AMP complex	*Rhodococcus sp.* USK13 DarR(K44A)-cAMP complex
**Data collection**				
Space group	$P3_221$	$P4_12_12$	C2	$P2_12_12$
PDB code	8SUA	8SV6	8SUK	8T5Y
Cell dimensions				
a, *b*, *c* (Å)	85.44,85.44,62.44	94.4,94.4,215.1	244.5,38.98,115.6	45.4,60.5,84.5
α,β,γ (°)	90.0,90.0,120.0	90.0,90.0,90.0	90.0,91.4,90.0	90.0,90.0,90.0
Resolution (Å)	47.72-1.60 (1.64-1.60)*	48.86-3.56 (3.60-3.56)	41.49-2.45 (2.52-2.45)	49.20-1.44 (1.46-1.44)
R_{sym}	0.047 (0.503)	0.141 (1.624)	0.107 (0.538)	0.054 (0.988)
R_{pim}	0.020 (0.406)	0.053 (0.561)	0.070 (0.378)	0.020 (0.433)
$I / \sigma I$	25.2 (2.0)	7.0 (1.5)	5.8 (1.1)	22.5 (2.0)
Completeness(%)	99.9 (99.0)	99.9 (99.9)	98.0 (88.2)	100.0 (99.9)
Redundancy	11.2 (11.1)	7.9 (7.4)	3.2 (2.6)	15.4 (11.7)
CC(1/2)	1.000 (0.805)	0.997 (0.357)	0.989 (0.654)	1.000 (0.743)
**Refinement**				
Resolution (Å)	47.72-1.60	48.86-3.56	41.49-2.45	49.20-1.44
No. reflections	35029 (2647)	12397 (1239)	40140 (2175)	543824 (2153)
R_{work} / R_{free} (%)	18.5/20.2	27.5/30.5	21.7/25.9	18.6/19.7
R.m.s. deviations				
Bond lengths (Å)	0.013	0.003	0.008	0.006
Bond angles (°)	1.15	0.618	0.939	0.892
Ramachandran analyses				
Favored (%)	99.0	91.9	96.2	99.5
Disallowed (%)	0.00	0.00	0.00	0.00

*Values in parentheses are for highest-resolution shell.

Supplementary Table 2: Data collection and refinement statistics: DarR-DNA complexes

	M. baixiangningiae DarR-20 bp DNA complex	*Rhodococcus* sp. USK13 DarR-20 bp DNA complex
**Data collection**		
Space group	$P2_1$	$P6_5$
	8SVD	8SVA
Cell dimensions		
a, b, c (Å)	68.0,155.8,117.8	128.9,128.9,113.9
α, β, γ (°)	90.0,97.2,90.0	90.0,90.0,120.0
Resolution (Å)	47.45-3.49 (3.58-3.49)*	42.19-2.96 (3.03-2.96)
R_{sym}	0.095 (0.402)	0.163 (1.467)
R_{pim}	0.061 (0.252)	0.064(1.575)
$I / \sigma I$	5.2 (1.9)	8.9 (0.5)
Completeness (%)	98.4 (99.9)	99.7 (99.9)
Redundancy	3.4 (3.5)	7.3 (4.7)
CC(1/2)	0.996 (0.868)	0.998 (0.317)
**Refinement**		
Resolution (Å)	47.45-3.49	42.19-2.96
No. reflections	30500 (2100)	22523 (1477)
R_{work} / R_{free} (%)	26.8/28.4	23.9/27.6
R.m.s. deviations		
Bond lengths (Å)	0.004	0.013
Bond angles (°)	0.823	0.937
Ramachandran analyses		
Favored (%)	94.8	92.7
Disallowed (%)	0.0	0.0

*Values in parentheses are for highest-resolution shell.

SUPPLEMENTARY REFERENCES

1. Gautam, S., Mahapa, A., Yeramala, L., Gandhi, A., Krishnan, S., Kutti, V. Jr. & Chatterji, D. Regulatory mechanisms of c-di-AMP synthase from *Mycobacterium smegmatis* revealed by a structure: Function analysis. *Protein Sci.* **32**, e4568 (2023).
